# Incidence and factors associated with SARS-CoV-2 infection and re-infection among people experiencing homelessness in Toronto, Canada: A prospective cohort study

**DOI:** 10.1371/journal.pone.0319296

**Published:** 2025-02-28

**Authors:** Lucie Richard, Brooke Carter, Michael Liu, Rosane Nisenbaum, Stephen W. Hwang

**Affiliations:** 1 MAP Centre for Urban Health Solutions, Unity Health Toronto, Toronto, Ontario, Canada; 2 ICES Western, London Health Sciences Research Institute, London, Ontario, Canada; 3 Harvard Medical School, Boston, Massachusetts, United States of America; 4 Dalla Lana School of Public Health, University of Toronto, Toronto, Ontario, Canada; 5 Applied Health Research Centre, Unity Health Toronto, Toronto, Ontario, Canada; 6 ICES Toronto, Toronto, Ontario, Canada; 7 Department of General Internal Medicine, University of Toronto, Toronto, Ontario, Canada; Bangladesh Open University, BANGLADESH

## Abstract

People experiencing homelessness are at elevated risk of SARS-CoV-2 infection, yet estimates generally exclude re-infections and rely on data sources affected by testing policies or study timing. In this prospective cohort study, we report incidence of SARS-CoV-2 infection and re-infections over time using a combination of community-based and study-administered testing, and assessed individual and housing-related factors associated with new infection. Individuals experiencing homelessness were randomly selected from 62 sites across Toronto, Canada, between June and September 2021. Participants provided detailed surveys and biological samples to test for SARS-CoV-2 (by RT-PCR and ELISA) every three months for one year. Self-reported data were verified and augmented through linkage to health administrative databases. Among 640 participants who completed 2,401 interviews, we identified 613 SARS-CoV-2 infection events, representing an incidence rate of 35.3 infections/100-person years (95% CI 31.6-39.4) prior to the onset of Omicron and 97.2 infections/100 person-years (95% CI 86.8-108.8) after Omicron. Nearly 30% (n = 182) of these events were re-infections. In multivariable models, post-Omicron interviews (adjusted rate ratio [aRR] 3.54 [95% CI 3.12-4.02]), history of prior COVID-19 infection (1 infection aRR 2.55 [95% CI 2.29-2.83]; 2 + infections aRR 2.28 [95% CI 1.80-2.89]) and residing in high- or moderate-exposure risk (congregate and shared) housing settings (high-exposure aRR 1.74 [95% CI 1.43-2.11]; moderate-exposure aRR 1.39 [1.15-1.68]) were most significantly associated with new infection. Our findings highlight that existing reports significantly underestimate SARS-CoV-2 infection burden among people experiencing homelessness, but confirms previously reported factors associated with infection, including congregate and shared housing settings. Reducing reliance on overcrowded emergency housing is necessary to reduce infection incidence in this population as well as associated inequities in downstream acute and chronic complications.

## Introduction

Homelessness and housing insecurity are major public health issues in Canada, with 3% of Canadians experiencing homelessness in their lifetime [1]. Temporary emergency housing for this population is often shared, crowded and subject to high turnover [2], all of which create environments where it is difficult or impossible to physically distance. As a result, people experiencing homelessness have been found to be at heightened risk of contracting severe acute respiratory syndrome coronavirus 2 (SARS-CoV-2) in most settings [3–11], and to have increased risk for adverse outcomes following SARS-CoV-2 infection [10].

Yet, few reliable estimates of longitudinally-ascertained infection incidence exist for this population [12–13]. Instead, most research falls into one of two categories: cross-sectional analyses in specific settings (e.g., shelters) using study-administered PCR or serologic assay testing [3–5,7,14–17], or larger-scale analyses reliant on administrative registries of community-based PCR testing [10,11,18,19]. The former yielded good, but highly variable estimates, emphasizing the importance of local context and study timing. The latter group of studies had broader representativeness, but were sensitive to testing policies [20–22] and willingness of individuals to be tested [23], leading to potential underestimation of infection or unrepresentative findings [24]. Our interim report [12] on incidence of first SARS-CoV-2 infection among people experiencing homelessness showed this population had a high rate of incident infection, but also confirmed that timing does strongly influence incidence estimates and most infections were only identified through study-administered testing.

Our interim analysis did not cover the full observation period, and did not account for re-infections, which are now recognized to be extremely common in this population [25]. Re-infections are also associated with increased risk for acute and chronic complications that substantially reduce quality of life, such as post-COVID condition, and are therefore important to quantify [26–28].

In the present study, we report the incidence of SARS-CoV-2 infection and re-infections in a representative cohort of longitudinally-followed people experiencing homelessness in Toronto, Canada. Using a combination of community-based and study-administered testing, we provide the most comprehensive and contemporary estimate of infection incidence for this population, and also assessed individual and housing-related characteristics associated with new infections.

## Methods

### Study design and setting

We conducted this prospective cohort study in Toronto, Ontario, a city in Canada on Treaty 13 territory, using data collected from the *Ku-gaa-gii pimitizi-win* study. *Ku-gaa-gii pimitizi-win*, which translates in English to “life is always/forever moving”, is a spirit name given in ceremony by Elder Dylan Courchene from Anishnawbe Health Toronto. The study protocol for the *Ku-gaa-gii pimitizi-win* study is available elsewhere [29]. Participant data were further linked and analyzed using unique encoded identifiers at ICES [30], an independent, non-profit research institute whose legal status under Ontario’s health information privacy law allows it to collect and analyze health care and demographic data, without consent, for health system evaluation and improvement.

In 2022, at least 20,700 individuals experienced homelessness in Toronto [31]. The 2021 point-in-time count indicated that 90% of homeless individuals in Toronto were sheltered in emergency and transitional accommodations [32]. In response to the pandemic, the city of Toronto implemented a comprehensive series of infection prevention strategies to protect residents experiencing homelessness, including enhanced infection prevention and control, routine screening and testing at shelters, opening of infection recovery sites, and moving thousands of individuals to temporary non-congregate shelters (‘physical distancing hotels’)[33]. The city and local health unit also strongly promoted COVID-19 vaccination once these became available in Ontario, resulting in 80% of our cohort having at least partial vaccination coverage by summer 2021 [34].

At the time of recruitment (June 16 to September 9 2021), the Delta variant (B.1.617.2) was rapidly replacing the Alpha variant (B.1.1.7) in COVID-19 infections in Toronto [35]. By December 2021 (approximately half-way through the study), Omicron variant BA.1 replaced Delta, increasing from < 1% to > 95% of infections over the month [36]. Additional Omicron variants predominated in large waves of activity over the remainder of the study period [35].

### Data sources

We used *Ku-gaa-gii pimitizi-win* data to establish the study cohort and to ascertain sociodemographic and housing information. We also accessed a number of databases at ICES (between 01/04/2023 and 01/09/2023) to cross-reference self-reported COVID-19 test and vaccination information, as well as provide information on comorbidities, and healthcare utilization. Administrative databases are described in detail in Supplement A in S1 File, and include: the ICES Registered Persons Database; the Discharge Abstract Database; the National Ambulatory Care Reporting System database; the Ontario Mental Health Reporting System database; the Ontario Health Insurance Plan (OHIP) claims database; the Community Health Centre database; the Ontario Cancer Registry; the COVID19 Integrated Testing Database; the Ontario COVID-19 Vaccine Database; and several ICES‐derived population‐surveillance databases, including the Ontario Asthma Database, the Chronic Obstructive Pulmonary Disease Database, the Ontario Diabetes Database, the Congestive Heart Failure Database, the Ontario Hypertension Database, and the Ontario HIV database.

### Recruitment and follow-up

We recruited individuals by random number schedule assigned to beds, rooms or tents at 62 participating sites (shelters, physical distancing hotels or encampments) in Toronto, Ontario between June 16 and September 9, 2021. Participants were eligible if they provided informed consent, were experiencing homelessness, were 16 years of age or older, were not already recruited into the study, and were willing to conduct follow-up interviews. To be included in this particular analysis, participants also had to consent and be successfully linked to ICES health administrative databases.

Participants were re-contacted for follow-up at three, six, nine and twelve months, with the final interview conducted on October 24, 2022. Each participant’s observation period covered up to 5 intervals, representing the following periods: baseline (March 1 2020 to baseline interview), 3-months (baseline to 3-month interview), 6-months (latest of baseline or 3-month interview to 6-month interview), 9-months (latest of baseline, 3- or 6-month interview to 9-month interview) and 12-months (latest of baseline, 3-, 6- or 9-month interview to 12-month interview). Observation ended at the final interview. Details regarding recruitment and follow-up procedures are available in the study protocol [29].

### Covariates

We obtained variables believed to influence infection risk through interview surveys, including sociodemographic information (age, gender, race, citizenship status, immigration history, education level), self-reported history of SARS-CoV-2 positive test(s), and activities and behaviors related to COVID-19 (e.g., masking; COVID-19 vaccination). Participants also provided self-reported housing history [37], which was used to create housing-related exposure variables that may increase risk for infection, such as number of moves during an interval, average number of people who shared participant living space, and proportion of time spent in various housing types. We also categorized housing types into high, moderate or low risk for infection based on whether the setting was congregate and/or crowded. “High exposure” settings refer to congregate homeless shelters, recovery centres, nursing homes, jails or immigration detention centers; “Moderate exposure” settings refer to transitional housing, rooming houses, encampments, being on the street, in rehab, hospital or ‘Other’ unclassifiable settings; and “Low exposure” settings included stays in own home, in supportive housing, a privately acquired hotel/motel, or doubling up with friends and/or family. Detailed definitions about exposure risk and other study covariates are provided in Supplement B in S1 File.

Finally, we obtained health-related characteristics from administrative databases, including past diagnoses of chronic conditions (e.g., hypertension, diabetes, asthma, chronic lung disease, chronic heart disease), recent healthcare for mental health or substance use, overall and by subtype (psychotic disorders including schizophrenia, substance use, mood and anxiety disorders, OCD/personality disorders or intentional self-injury); history of positive PCR tests or COVID-19 vaccines; and healthcare utilization in the past year including acute care admissions, emergency department visits and outpatient visits.

### Outcomes

Our primary outcome was new SARS-CoV-2 infection (first infection or re-infection) at the end of an interval, defined as the period between two interviews. We ascertained infection through a combination of sources to provide the most robust and comprehensive possible measure, as people routinely experience infection without their knowledge (and therefore may not be tested)[4], anti SARS-CoV-2 antibodies decay over time [38], and self-report can suffer from recall bias or social desirability bias, particularly among populations experiencing significant stigma [39].

Specifically, we defined new infections as any of the following: self-reported positive polymerase chain reaction (PCR) or rapid antigen (RAT) test; positive PCR test reported in the COVID-19 Integrated Testing Database; positive study-administered PCR test [40] from the interview saliva sample; or positive evidence of at least two of three anti SARS-CoV-2 antibodies from study-acquired blood samples tested by enzyme-linked immunosorbent assay [41]. Where vaccinated participants had positive serology without sufficiently elevated anti-nucleocapsid protein, we deemed them uninfected unless they also had evidence of a positive PCR or RAT test, as COVID-19 vaccines approved in Canada increase antibodies aside from anti-nucleocapsid protein and thus these antibodies cannot be independently used as a surrogate infection measure [41].

New infections following a history of initial infection (re-infections) were defined as positive PCR or RAT tests occurring more than 90 days after the last infection began; positive serology results where participants had previously sero-reverted; or anti-nucleocapsid protein levels that increased above the assay’s coefficient of variation after a downward trend in anti-nucleocapsid levels had been previously established. A detailed report of the method for comprehensive identification of re-infections is available elsewhere [25].

### Statistical analysis

We present baseline characteristics of study participants overall and by successful linkage to ICES (in Supplement C, table 1 in S1 File), as well as by outcome (in Supplement C, table 2 in S1 File). We further provide outcome rates per 100 person-years by reporting period (before or after Omicron variants became dominant), as the emergence of Omicron variants has been documented to be more infectious than preceding variants [42]. Where participants experienced new infection without precise date information available, observation time was stopped at a randomly assigned day between the prior and current interviews [43]. Confidence intervals (CI) for rates and rates per 100 person-years were calculated using the Wilson Score method for proportions.

Finally, as housing and behavioral characteristics were assessed at each interview and could thus vary over time, factors associated with new infection were evaluated on interval-level data using unadjusted and adjusted rate ratios (uRR and aRR) and 95% confidence intervals estimated from modified Poisson regression models with generalized estimating equations (to account for the correlated nature of repeated measures), offset by the log of total observation time. Variables with statistically significant associations in unadjusted models were included in a multivariable modified Poisson regression model, unless correlation coefficients (estimated using variance inflation factor and polychoric correlation) with other relevant covariates were too high (defined as coefficients of greater than 2.5 and 0.4, respectively).

As all variables had little to no missingness (less than 5% and in most cases none or less than 1%), we reported such groups in their own category to present complete-case results without imputation, in line with recommendations [44]. Throughout, we defined statistical significance using p-values of <  0.05 (2-sided), and suppressed cells <= 5 to protect participant privacy. All analyses were conducted using SAS enterprise guide v8.3 (SAS Institute Inc., Cary, NC, USA).

### Ethical review

This study received ethics approval from the Research Ethics Board at Unity Health Toronto (REB# 20-272), and follows the Reporting of Studies Conducted Using Observational Routinely Collected Data (RECORD) reporting guidelines (Supplement D in S1 File) [45]. Participants were all adults or emancipated minors, and provided written informed consent.

## Results

**Fig 1:**
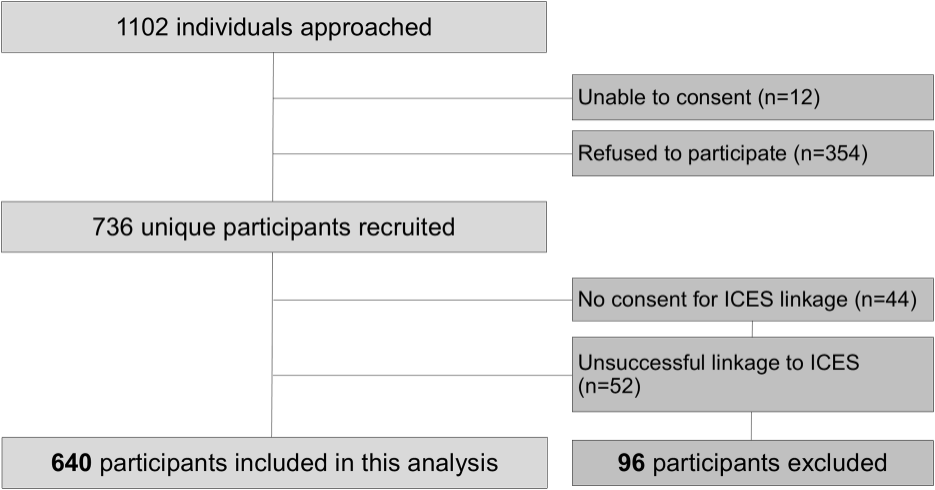
Ku-gaa-gii pimitizi-win recruitment, reasons for non-participation or exclusion from analysis.

Of the 1102 individuals that were randomly approached during recruitment efforts, 354 declined to participate and 12 were unable to consent (Fig 1). Of the overall 736 participants who were recruited into the study, 44 individuals refused the additional consent for linkage to ICES, and 52 were not successfully linked, resulting in a final sample of 640 participants for this analysis, or 86.9% of the overall *Ku-gaa-gii pimitizi-win* cohort. Supplement C, table 1 in S1 File compares characteristics of participants who were successfully linked to ICES compared to the cohort overall: individuals successfully linked were very similar to the overall cohort, except for a somewhat lower rate of refugees and persons with temporary or other legal status. A majority of participants were 30–49 years old (44.3%) or 50–69 years old (35.7%), self-reported being male (67.4%), White (48.8%), and Canadian citizens (76.6%).

Among participants, 81 (16.7%) were lost to follow-up after baseline; 71 (11.1%) provided one follow-up interval of data; 75 (11.7%) provided two follow-up intervals of data; 112 (17.5%) provided three follow-up intervals; and 301 (47.0%) provided all four follow-up interviews. Overall, participants contributed a total of 2,401 interviews, representing 883.9 person-years of observation time pre-Omicron and 309.8 person-years of observation time post-Omicron.

SARS-CoV-2 infection and associated outcome rates pre- and post-Omicron are presented in Table 1. A total of 613 infections were detected across 2,401 intervals, of which 312 were identified during interviews before Omicron became prevalent and 301 identified during interviews after Omicron became prevalent. This represents an infection incidence rate of 35.3 (95% CI 31.6–39.4) infection events/100 person-years pre-Omicron and 97.2 (95% CI 86.8–108.8) infection events/100 person-years post-Omicron. 29.7% of infection events were re-infections, with a majority of re-infections (n = 127) occurring post-Omicron. Overall, by the end of the observation period, 419 (65.5%) participants overall had evidence of at least one SARS-CoV-2 infection; 25% of these had at least two infection events.

**Table 1:** SARS-CoV-2 infections among Ku-gaa-gii pimitizi-win participants, by period.

	Follow-up pre-Omicron^1^	Follow-up post- Omicron^2^
**SARS-CoV-2 infection events, N (%)**	312 (26.6%)	301 (24.5%)
**Infection event rate/100 person-yrs (95% CI)**	35.3 (31.6–39.4)	97.2 (86.8–108.8)
**Re-infection events, N (%)**	55 (4.7%)	127 (10.8%)
**Participants with 1** + **event by end of observation, N (%)**	419 (65.5%)	
**Participants with 2** + **events by end of observation, N (%)**	163 (25.5%)	

CI=Confidence Interval. 95% confidence interval calculated using the Wilson Score method for proportions

^1^1172 interviews overall occurred before December 31 2021

^2^1229 interviews overall occurred after or on December 31 2021

Table 2 shows the results of unadjusted models assessing factors potentially associated with new infection. Timing of interviews was strongly associated with infection, with interviews during the post-Omicron period were more than three times more likely to reveal a new infection than earlier interviews (unadjusted rate ratio [uRR] 3.40 [95% CI 3.00–3.84]). A number of other factors were also positively associated with infection, including: Black (uRR 1.28 [95% CI 1.10–1.49]) or other racialized (uRR 1.20 [95% CI 1.01–1.43]) self-reported race category; Immigration to Canada (>10 yrs ago uRR 1.18 [95% CI 1.02–1.36]); Cancer diagnosis in the past ten years (uRR 1.38 [95% CI 1.07–1.80]); Obsessive compulsive or other personality disorder (uRR 1.55 [95% CI 1.20–2.00]); Number of SARS-CoV-2 infections at baseline (1 infection uRR 2.37 [95% CI 2.12–2.65]; 2 + infections uRR 2.00 [95% CI 1.47–2.73]); and a number of environmental factors, such as proportion of interval residing in congregate shelters (per 10% increase in time, uRR 1.04 [95% CI 1.02–1.05]) and spending their largest share of time residing in high-exposure settings (uRR 1.50 [95% CI 1.22–1.86]). COVID-19 vaccination status at baseline was not associated with infection risk.

**Table 2:** Unadjusted modified Poisson regression with generalized estimating equations assessing each factor potentially associated with SARS-CoV-2 infection by the end of an interval (2,401 intervals overall).

Participant characteristics	uRR^a^	95% CI	P-value
Age category (ref = 30–49 years old)			
16–29 years old	0.96	0.77–1.21	0.74
50–69 years old	0.94	0.81–1.09	0.40
70 + years old	1.05	0.83–1.33	0.67
Self-reported gender (ref=Male)			
Female	0.86	0.74–1.00	0.05
LGBTQS2 + /Non-binary/Other	0.65	0.37–1.14	0.13
Self-reported race category (ref=White)			
Black	1.28	1.10–1.49	0.001
Indigenous	1.10	0.81–1.51	0.54
Other/multiracial	1.20	1.01–1.43	0.04
Refused/Don’t know	0.93	0.68–1.28	0.67
Citizenship status (ref=Citizen)			
Landed immigrant/permanent resident	1.14	0.96–1.35	0.12
Refugee claimant	1.09	0.81–1.48	0.56
Temporary status/Other	1.12	0.80–1.59	0.51
Immigration history (ref=Born in Canada)			
Immigrated to Canada > 10 years ago	1.18	1.02–1.36	0.03
Immigrated to Canada <= 10 years ago	1.19	0.99–1.43	0.06
Education completed (ref=Any post-secondary)			
Less than high school	1.13	0.96–1.33	0.14
High school or equivalent	1.10	0.94–1.28	0.25
**Health at baseline**	**uRR** ^a^	**95% CI**	**P-value**
Hypertension (ref=No)	1.00	0.84–1.19	0.99
Diabetes (ref=No)	0.99	0.81–1.22	0.95
Asthma (ref=No)	0.97	0.82–1.14	0.68
Chronic Lung Disease (ref=No)	0.95	0.78–1.17	0.65
Chronic Heart Disease (ref=No)	0.97	0.71–1.32	0.84
History of Stroke (ref=No)	1.05	0.75–1.46	0.78
Chronic Kidney Disease (ref=No)	0.99	0.59–1.69	0.98
Chronic Neurological Disorder (ref=No)	0.97	0.73–1.28	0.83
Liver Disease (ref=No)	1.12	0.82–1.52	0.49
Cancer diagnosis within the past ten years (ref=No)	1.38	1.07–1.80	0.01
HIV/AIDS (ref=No)	0.98	0.56–1.71	0.94
Any mental health or substance use related disorder (ref=No)			
Any	0.96	0.84–1.09	0.50
Substance use disorders	1.06	0.90–1.26	0.48
Psychotic disorders including schizophrenia	1.08	0.88–1.34	0.45
Mood and anxiety disorders	0.94	0.79–1.12	0.47
OCD/Personality disorders	1.55	1.20 – 2.00	0.001
Intentional self-injury	0.94	0.64–1.39	0.77
Number of COVID-19 infections at baseline (ref=Zero)			
1 infection	2.37	2.12–2.65	<.001
2 + infections	2.00	1.47–2.73	<.001
COVID-19 vaccines before baseline interview (ref=Full primary series)			
None	1.08	0.93–1.26	0.33
Incomplete primary series	1.04	0.87–1.24	0.68
**Health-related behaviours during interval**	**uRR** ^a^	**95% CI**	**P-value**
Paid or volunteer work (ref=No)	1.00	0.87–1.16	0.95
Alcohol consumption (ref=No)	1.05	0.90–1.21	0.54
Tobacco consumption (ref=Yes)	0.83	0.72–0.95	0.01
Illegal/prescription medication consumed for non-medical reasons (ref=No)	0.96	0.83–1.11	0.61
PHG #1: wears face mask in public (ref=Good [Often or always])			
Poor (Never, Rarely or Occasionally)	1.13	0.95–1.34	0.16
PHG #2: distances in public places (ref=Good [Often or always])			
Poor (Never, Rarely or Occasionally)	1.09	0.91–1.31	0.35
PHG #3: avoids crowded places or gatherings (ref=Good [Often or always])			
Poor (Never, Rarely or Occasionally)	1.08	0.91–1.27	0.39
Interview reported after Omicron variants became dominant (ref=No)	3.40	3.00–3.84	<.001
**Housing-related exposure during interval**	**uRR** ^a^	**95% CI**	**P-value**
Proportion of interval spent in:			
Congregate shelter (every 10% increase)	1.04	1.02–1.05	<.001
Non-congregate shelter (every 10% increase)	0.97	0.94–0.99	0.01
Physical distancing hotel (every 10% increase)	0.99	0.97–1.00	0.10
Own place (every 10% increase)	1.01	0.98–1.03	0.53
Staying with friends or family (every 10% increase)	0.94	0.90–0.98	0.004
High Exposure^b^ setting (every 10% increase)	1.04	1.02–1.05	<.001
Moderate Exposure^c^ setting (every 10% increase)	0.97	0.96–0.99	<.001
Low Exposure^d^ setting (every 10% increase)	0.99	0.97–1.01	0.36
Majority of interval spent in (ref=Low Exposure^d^ setting)			
High Exposure^b^ setting	1.50	1.22–1.86	<.001
Moderate Exposure^c^ setting	1.09	0.88–1.34	0.43
Number of moves during period	1.04	0.99–1.09	0.10
Average number of people who shared living space	1.01	1.00–1.01	0.001

CI=Confidence Interval; PHG=Public Health Guideline

^a^Unadjusted rate ratio estimated using modified Poisson regression. Models were fitted with generalized estimating equations to account for the correlated nature of interval-level responses.

^b^’High exposure’ includes time residing in a congregate homeless shelter, recovery centre, nursing home, jail or immigration detention center

^c^’Moderate exposure’ includes time residing in a physical distancing hotel, non-congregate shelter, transitional housing, rooming house, encampment, on the street, rehab, hospital or ‘other’ settings.

^d^’Low exposure’ includes time residing in own home, supportive housing, private hotel/motel, or staying with friends and family

Correlation results (not shown) demonstrated that immigration was significantly correlated with race category, and most of the significant environmental factors were strongly collinear. Therefore, we selected self-reported race category and level of housing-related exposure risk to include in the multivariable model.

Table 3 presents the joint associations between selected factors and new infection at an interview. Few covariate associations were attenuated after adjustment. Interview after Omicron remained strongly associated with new infection (aRR 3.54 [95% CI 3.12–4.02]), as was history of COVID-19 infection at baseline (1 infection aRR 2.55 [95% CI 2.29–2.83]; 2 + infections aRR 2.28 [95% CI 1.80-2.89]) and spending their largest share of time residing in high- or moderate-exposure housing (High-exposure housing aRR 1.74 [95% CI 1.43–2.11]; Moderate-exposure housing 1.39 [95% CI 1.15–1.68]). Additional covariates significantly associated with new infection included self-reported race category (Black aRR 1.37 [95% CI 1.20–1.55]; Other racialized aRR 1.14 [95% CI 1.00–1.31]); Cancer diagnosis (aRR 1.58 [95% CI 1.39–1.78]); and obsessive compulsive or other personality disorder (aRR 1.69 [95% CI 1.32–2.18]).

**Table 3:** Multivariable modified Poisson regression with generalized estimating equations assessing factors associated with SARS-CoV-2 incident infection by the end of an interval (2,401 intervals overall).

	aRR^a^	95% CI	P-Value
Interview reported after Omicron variants became dominant (ref=No)	3.54	3.12–4.02	< 0.001
Self-reported race category (ref=White)			
Black	1.37	1.20–1.55	< 0.001
Indigenous	1.00	0.78–1.27	0.98
Other/multiracial	1.14	1.00–1.31	0.05
Refused/Don’t know	1.03	0.74–1.43	0.88
Cancer diagnosed in the past ten years (ref=No)	1.58	1.39–1.78	< 0.001
OCD/Personality Disorder at baseline (ref=No)	1.69	1.32–2.18	< 0.001
Number of COVID-19 infections at baseline (ref=None)			
1 infection	2.55	2.29–2.83	< 0.001
2 + infections	2.28	1.80–2.89	< 0.001
Top housing type during interval (ref=Low-exposure^b^)			
High-exposure^c^	1.74	1.43–2.11	< 0.001
Moderate-exposure^d^	1.39	1.15–1.68	< 0.001

^a^Adjusted rate ratio estimated using multivariable modified Poisson regression. Models were fitted with generalized estimating equations to account for the correlated nature of interval-level responses.

^b^’Low exposure’ includes time residing in own home, supportive housing, private hotel/motel, or staying with friends and family.

^c^’High exposure’ includes time residing in a congregate homeless shelter, recovery centre, nursing home, jail or immigration detention center.

^d^’Moderate exposure’ includes time residing in a physical distancing hotel, non-congregate shelter, transitional housing, rooming house, encampment, on the street, rehab, hospital or ‘other’ settings.

## Discussion

In this representative cohort of people experiencing homelessness in Toronto, Canada, 65.5% of participants had at least one SARS-CoV-2 infection by the end of observation, and more than 25% had two or more infection events. Overall, 35.3 infection events/100 person-years occurred prior to Omicron, and 97.2 events/100 person-years occurred post-Omicron. New infection risk was higher among individuals who self-identified as Black, as non-Indigenous visible minorities or as multiracial, and among people residing in housing deemed to be high- or moderate- exposure risk by virtue of it being crowded and/or congregate.

To our knowledge, only two longitudinal studies have measured SARS-CoV-2 incidence in this population using study-administered testing. One was our interim report [12], which assessed incidence of first SARS-CoV-2 infections in the first 6-months of the *Ku-gaa-gii pimitizi-win* study, and the other was a French cohort study [13] that determined cumulative incidence through seroprevalence measured twice, three months apart. Both of these studies did not measure incidence including re-infections, and the French study was conducted prior to the emergence of Omicron variants.

Other literature about COVID-19 infection rates among people experiencing homelessness have mostly been cross-sectional in nature [3–5,7,14–17] or leveraged administrative registries of community-based PCR testing [10,11,18,19]. The cross-sectional analyses were mostly completed in the early phases of the pandemic, were quite sensitive to study timing and local context, and those leveraging serology were, by design, unable to report on re-infections. The studies based on administrative data were, by contrast, highly sensitive to local testing policies and generally only captured a small fraction of infection events [12], which could also result in biased estimates wherever local testing was limited by policies [20–22] or individual testing-related behaviours [23]. For example, a study in our region and approximate time frame using administrative data [11] estimated a maximum of 25 infections/100 person-years in this population (during the earliest Omicron wave), which is nearly four times less than our observed Omicron-era incidence rate of 97.2 events/100 person-years. Thus, alternative methodologies have generally presented an incomplete or drastically underestimated picture of SARS-CoV-2 incidence in this population, and our findings provide the most robust and comprehensive estimate of SARS-CoV-2 incidence encompassing re-infections in a cohort of people experiencing homelessness.

Another comparison group is available using the general population, through Canadian population-based seroprevalence studies summarized by the Canadian COVID-19 Immunity Task Force [46]. By the end of October 2022 (when our study observation ended), Ontario seroprevalence estimates of infection-induced antibodies averaged around 64% (95% CI 60.9-67.0%) [46], approximating rates of prevalence of any infection in our cohort. Thus, in line with our interim report [12], differences in history of any SARS-CoV-2 infection have continued to narrow in the post-Omicron era. However, this comparison does not account for re-infections [26], which by definition cannot be measured in cross-sectional seroprevalence studies. Unfortunately, there is no appropriate comparison for our re-infection estimate, as we uniquely incorporate evidence from repeated serology testing [25]. However, our previous study [25] did estimate that 9% of the cohort had at least one re-infection identified without serology, which was nearly triple that of pooled estimates from Omicron-era re-infection studies in the general population [47]. Therefore, although people experiencing homelessness may now have similar levels of overall history of any infection, the total number of infection events strongly suggests that important disparities remain in the rates of re-infection and thus overall COVID-19 infection burden.

Our finding that infection risk was higher among individuals who self-identified as Black, as non-Indigenous visible minorities or as multiracial is consistent with existing literature [48,49], which links this disparity to structural racism, including higher exposure to the virus through work settings and/or overcrowded living environments [48], or decreased access to clinical care [49]. In our study, living environment and engaging in paid or unpaid work did not explain heightened risk in these groups. However, it is possible that the type of work-related exposure [50] (not measured in our study) may have contributed to observed differences.

Finally, the significant association between crowded and congregate housing and new infection is also consistent with previous literature [4,6,8,12,51]. While the city of Toronto made significant efforts to enhance emergency shelter system infection prevention and control measures during the pandemic [33] we still observed evidence of very high infection transmission. This suggests that such measures on their own are insufficient to mitigate against risks inherent in congregate and crowded settings, which is consistent with recent simulation and intervention work [52,53]. What did appear to reduce risk was providing lower-risk housing settings, such as physical distancing hotels (which remain “moderate-risk”, but were a significant improvement over the congregate shelters from which individuals were moved), or exiting homelessness itself (to mostly ‘low-risk’ settings like permanent or supportive housing).

Housing is clearly a modifiable risk factor, and our findings support the conclusion that reductions in crowding and separate rooms in shelters may be the most effective approach to protect individuals experiencing homelessness from infection. Unfortunately, the pandemic-related funding for hotels and similar distancing programs in Toronto were a one-time, time-limited initiative, and funding ran out shortly after the end of this study’s observation period. These programs have now mostly ceased operations [54,55] and returned to offering pre-pandemic forms of emergency shelter [55] which, despite continuation of enhanced infection prevention and control efforts, is likely to result in increased infection transmission moving forward. Given the known risks of downstream acute and chronic issues associated with SARS-CoV-2 infection and re-infection [26–28], this will continue to be a major health equity issue. All three levels of government in Canada should recognize their shared responsibility in providing safe shelter that reflects the ongoing presence and endemicity of SARS-CoV-2 (as well as other infectious diseases). In particular, policymakers should pivot funding towards the provision of specifically non-congregate shelter services, ideally with minimum space requirements to avoid overcrowding.

### Limitations

Our study followed a cohort of people experiencing homelessness randomly sampled from sites across Toronto and ascertained infection through a combination of methods, ensuring robust and representative results for the region. However, relatively few individuals were recruited from unsheltered settings, which comprise approximately 10% of Toronto’s unsheltered homeless population [32]. Thus, our results approximate, but are not fully representative of, all people experiencing homelessness in Toronto. Additionally, our survey and housing data were self-reported. While interviews occurred every three months to reduce the potential for unreliable reports and we supplemented with study-administered testing and administrative data wherever possible, self-reported data are susceptible to recall or social desirability bias, which can be particularly important among populations facing significant stigma [39].

## Conclusions

People experiencing homelessness in Toronto in 2021 and 2022 had very high SARS-CoV-2 infection incidence, and particularly high re-infection rates. Findings suggest that existing literature significantly underestimates the burden of COVID-19 infection in this population. Findings also support the conclusion that upstream structural risks and reliance on congregate shelter settings render certain unhoused individuals particularly vulnerable, with less crowded and private shelter options associated with lower infection risk.

Future efforts to prevent COVID-19 infection as well as other infectious disease transmission in this population should focus on prioritizing non-congregate and less crowded shelter systems.

## Supporting information

S1 File Supplementary Materials: Supplements A through D Contains the description of administrative sources (A), variable definitions (B), supplementary tables (C), and RECORD statement (D).
(DOCX)
