# Supplementary material for: Incidence and factors associated with SARS-CoV-2 infection and re-infection among people experiencing homelessness in Toronto, Canada: A prospective cohort study
Source: PLoS One. 2025 Feb 28;20(2):e0319296. doi: 10.1371/journal.pone.0319296 (PMC11870375; doi:10.1371/journal.pone.0319296)
Supplement: S1 File — Supplementary Materials: Supplements A through D Contains the description of administrative sources (A), variable definitions (B), supplementary tables (C), and RECORD statement (D). (DOCX) [file pone.0319296.s001.docx]

**Supplementary Materials**

**Supplement A – Description of administrative data sources**

| Data source | Description |
| --- | --- |
| **ICES Registered Persons Database (RPDB)** | The RPDB database provides demographic information on any individual who has ever received an Ontario health card number, including date of birth and death (if applicable), sex-at-birth, and postal code for each year. |
| **CIHI Discharge Abstract Database (DAD)** | DAD captures administrative (institution-hospital number, admission category, length of stay, disposition), clinical (diagnoses, procedures, physician) and demographic information (patient gender, date of birth, postal code, county and residence code), on hospital discharges including deaths, sign-outs and transfers. |
| **National Ambulatory Care Reporting System (NACRS)** | NACRS captures information on patient visits to hospital and community based ambulatory care such as day surgery, outpatient clinics and emergency departments within Ontario. |
| **Ontario Mental Health Reporting System (OMHRS)** | OHMRS collects data on patients in adult designated inpatient mental health beds. |
| **Ontario Health Insurance Plan (OHIP) claims** | The OHIP claims database contains most claims paid for by the Ontario Health Insurance Plan. The data covers all health care providers who can claim under OHIP (this includes physicians, groups, laboratories, and out-of-province providers) for the purposes of maintaining a record of the patient and physician, services provided, date of the service, associated diagnosis, and fees paid. Excludes services provided by Community Health Centres. |
| **Community Health Centre database (CHC)** | CHC captures services provided in Community Health Centres, which provide primary health and health promotion programs for individuals, families and communities particularly those underserved or who have barriers to accessing other forms of primary care. Provides information on CHC patients, services, physicians and other allied healthcare professionals. |
| **Ontario Cancer Registry (OCR)** | OCR is the provincial database of information for all Ontario residents who have been diagnosed with or who have died of cancer. |
| **Ontario COVID-19 Integrated Testing Database (C19INTGR)** | C19INTGR a comprehensive dataset of all available COVID-19 diagnostic laboratory results in Ontario to support efforts to curb the pandemic. Includes data from the Ontario Laboratories Information System (OLIS), distributed testing data from laboratories (DL) within the COVID-19 Diagnostic Network, and Public Health Case & Contact Management (CCM) Solution, formerly known as the integrated Public Health Information System (iPHIS) |
| **Ontario COVID-19 Vaccine Database (COVAXON)** | The Ontario COVID-19 vaccine database contains information on all COVID-19 vaccination events for the purpose of monitoring vaccination uptake. |
| **Ontario Asthma Database (ASTHMA)** | The Ontario Asthma Database is contains all Ontario asthma patients identified through a validated case definition^1^ since 1991 through primary care patient records and hospital administrative data. |
| **Chronic Obstructive Pulmonary Disease Database (COPD)** | The Ontario Chronic Obstructive Pulmonary Disease Database contains all Ontario COPD patients identified through a validated case definition^2^ since 1991 through primary care patient records and hospital administrative data |
| **Ontario Diabetes Database (ODD)** | The Ontario Diabetes Database contains all Ontario diabetes patients identified through a validated case definition^3^ since 1991 through primary care patient records and hospital administrative data. |
| **Congestive Heart Failure Database (CHF)** | The Ontario Congestive Heart Failure Database contains all Ontario individuals with CHF identified through a validated case definition^4^ since 1991 through primary care patient records and hospital administrative data. |
| **Ontario Hypertension Database (HYPER)** | The Ontario Hypertension Database contains all Ontario individuals with hypertension identified through a validated case definition^5^ since 1991 through primary care patient records and hospital administrative data |
| **Ontario HIV database (HIV)** | The Ontario HIV Database contains all Ontario HIV positive patients identified through a validated case definition^6^ since 1992. |
|  |  |

**Supplement B – Variable definitions**

The table below includes all variables included in this analysis. Covariates are either known from existing literature to affect risk for SARS-CoV-2 infection or are related to SARS-CoV-2 related adverse health outcomes (which may also, indirectly, affect behaviours affected risk of infection). In all instances, measures were collected based on a Settler Canadian (Western) understanding of health-related factors and prevention guidelines appropriate during the COVID-19 pandemic.

| **Variable** | Definition |
| --- | --- |
| Outcome | |
| **SARS-CoV-2 infection** | SARS-CoV-2 infection was ascertained at the end of each reporting interval through any of the following sources of evidence:  a) positive PCR or Rapid Antigen Test (RAT) self-reported by the participant;  b) positive PCR test reported in the COVID-19 Integrated Testing Database;  c) positive PCR test administered during an interview; or  d) at least two of three anti SARS-CoV-2 antibodies exceeding positivity thresholds in the blood sample taken during the interview.  For intervals where the participants already had a prior history of SARS-CoV-2 infection, we identified re-infections where  a) positive PCR or RAT tests (from any source) were found more than 90 days after the previous infection began,  b) positive serology results after prior sero-reversion, or  c) positive serology results where anti-Nucleocapsid protein levels increased sufficiently after a downward trend had been previously established, as documented in detail elsewhere.^7^ |
| Covariates | |
| **Age category** | Participant’s age as calculated from the ICES Registered Persons Database date of birth and cohort entry date. Expressed as age categories (‘16 to 29 years’; ‘30 to 49 years’; ‘50 to 69 years’; and ‘70+ years’). |
| **Gender** | Participant’s self-reported gender, as of the date of the baseline survey. Answers include ‘male’, ‘female’, ‘LGBTQS2+/Non-binary/Other’ (which also includes ‘gender queer’, ‘agender’, ‘transgender’ or similar) and ‘refused/don’t know’. |
| **Race category** | Participant’s self-reported race identity, as of the date of the baseline survey. Answers include ‘White’, ‘Black’, ‘Indigenous’ (including First Nations, Métis or Inuit), ‘Other/multiracial’ (which includes Arab/Middle Eastern/West Asian, Latin American, East or Southeast Asian, South Asian/Indo Carribean, or where the participant reported multiple racial categories), ‘refused/don’t know’ and ‘missing’. The ‘missing’ category was not included in modelling. |
| **Citizenship status** | Participant’s self-reported citizenship status, as of the date of the baseline survey. Answers include ‘Citizen’, ‘Landed immigrant’ (also known as ‘Permanent resident’), ‘Refugee’, ‘Temporary/Other’ and ‘Refused/Don’t know’. |
| **Immigration history** | Recentness of participant immigration to Canada, as of the date of the baseline survey. Calculated from the Citizenship status and year of immigration variables. Categories include ‘Born in Canada (n/a)’, ‘10 or more years ago’, and ‘Less than 10 years ago’. |
| **Education level** | Participant’s self-reported highest level of completed education, as of the baseline interview date. Answers include ‘less than high school’ (secondary school), ‘high school’, ‘any-post-secondary’ (which combines ‘vocational/technical school’, ‘college/university’, ‘graduate/professional school’), or ‘Refused/Don’t know’. |
| **COVID-19 vaccination at baseline** | Participant’s self-reported or COVAXON-confirmed receipt of COVID-19 vaccination by the baseline interview date. Options include ‘none’ (ie. Unvaccinated), ‘incomplete primary series’, or ‘complete primary series’. |
| **Hypertension** | Presence in the ICES Hypertension Database at any point prior to cohort entry. |
| **Diabetes** | Presence in the ICES Diabetes Database at any point prior to cohort entry |
| **Asthma** | Presence in the ICES Asthma Database at any point prior to cohort entry |
| **Chronic lung disease** | Presence in the ICES COPD Database or diagnosis by a physician for chronic lung disease (including emphysema or chronic bronchitis) at any point prior to cohort entry. |
| **Chronic heart disease** | Presence in the ICES CHF Database or any hospitalization or physician billing related to coronary artery disease or myocardial infarction at any point prior to cohort entry. |
| **History of stroke** | One hospitalization or 2 physician billings <1 yr apart within the past 5 years for healthcare related to a past stroke. |
| **Chronic kidney disease** | One hospitalization or 2 physician billings or ED visits <1 yr apart within the past year for healthcare related to chronic kidney disease. |
| **Chronic neurological disorder** | Presence in the Dementia database; or, any hospitalization, ED visit or physician billing in the past year relating to traumatic brain injury or Guillaine-Barre Syndrome; or, one hospitalization or at least 3 physician billings in the past year related to epilepsy; or, one hospitalization or at least 5physician billings over the past 2 years related to multiple sclerosis. |
| **Liver disease** | Any hospitalization, ED visit or physician billing in the past year for healthcare related to liver disease. |
| **Cancer** | Diagnosis in the Ontario Cancer Registry within 10 years of cohort entry. |
| **HIV/AIDS** | Presence in the ICES HIV Database at any point prior to cohort entry. |
| **Any mental health or substance use related disorder** | One hospitalization or at least 3 outpatient/ED visits in the past year for any mental health or substance use related concern. |
| **Substance use disorder** | One hospitalization or at least 3 outpatient/ED visits in the past year for substance use related concern. |
| **Psychotic disorders including schizophrenia** | One hospitalization or at least 3 outpatient/ED visits in the past year for psychotic disorder related concern, including schizophrenia. |
| **Mood/anxiety disorder** | One hospitalization or at least 3 outpatient/ED visits in the past year for mood or anxiety related concern |
| **OCD/personality disorder** | One hospitalization or at least 3 outpatient/ED visits in the past year for obsessive compulsive disorder or other personality disorder related concern |
| **Intentional self-injury** | Any hospitalization or ED visit in the past year related to self-harm or intentional self-injury |
| **Number of COVID-19 infections at baseline** | Number of SARS-CoV-2 infections identified at the baseline interview. Options include ‘none’, ‘1’ or ‘2+’. |
| **Paid or volunteer work** | Participant’s self-reported work experience during the interval. Work includes any form of paid activity, and volunteer work includes any form of unpaid activity on behalf of another person or organization. Answers categorized into ‘Yes’ and ‘No/Refused/Don’t know’. ‘Refused/don’t know’ category is not included in modelling. |
| **Alcohol consumption** | Consumption of alcohol by the participant during the interval, irrespective of frequency or quantity of consumption. Categories include ‘Yes’ and ‘No’. ‘Refused/don’t know’ category is not included in modelling. |
| **Frequency of alcohol consumption** | Average frequency of alcohol consumption by the participant during the interval, irrespective of quantity consumed. Categories include ‘Never’, ‘Monthly or less’, ‘2-4 times a month’, ‘2-3 times a week’ and ‘4+ times a week’. ‘Refused/don’t know’ category is not included in modelling. |
| **Tobacco consumption** | Consumption of tobacco by the participant during the interval, irrespective of frequency or quantity of consumption. Categories include ‘Yes’ and ‘No’. ‘Refused/don’t know’ category is not included in modelling. |
| **Frequency of tobacco consumption** | Average frequency of tobacco consumption by the participant during the interval, irrespective of quantity consumed. Categories include ‘Never’, ‘Less than daily’, and ‘Daily’. ‘Refused/don’t know’ category is not included in modelling. |
| **Consumption of illegal or prescription medication for non-medical reasons** | Consumption of any illegal drug or prescription medication taken for non-medical reasons during the interval. Categories include ‘No’ or ‘Yes’. Refused/don’t know category is not included in modelling. |
| **Observance of public health guidelines: wearing a face mask in public places** | The participant’s self-reported adherence to public health guidelines active in Ontario during the interval (specifically: the degree to which the participant wears a face mask when in public places). Answers include ‘Low’ (including Never/rarely/occasionally), ‘High’ (including ‘Often/Always’) and ‘Refused/Don’t know. ‘Refused/don’t know’ category is not included in modelling. |
| **Observance of public health guidelines: distancing in public places** | The participant’s self-reported adherence to public health guidelines active in Ontario during the interval (specifically: the degree to which the participant practices physical distancing while in public places). Answers include ‘Low’ (including Never/rarely/occasionally), ‘High’ (including ‘Often/Always’) and ‘Refused/Don’t know. ‘Refused/don’t know’ category is not included in modelling. |
| **Observance of public health guidelines: avoiding crowded places or gatherings** | The participant’s self-reported adherence to public health guidelines active in Ontario during the interval (specifically: the degree to which the participant avoids crowded places or gatherings). Answers include ‘Low’ (including Never/rarely/occasionally), ‘High’ (including ‘Often/Always’) and ‘Refused/Don’t know. ‘Refused/don’t know’ category is not included in modelling. |
| **Observance of public health guidelines: washing hands several times per day** | The participant’s self-reported adherence to public health guidelines active in Ontario during the interval (specifically: the degree to which the participant washes their hands with soap/hand sanitizer several times per day). Answers include ‘Low’ (including Never/rarely/occasionally), ‘High’ (including ‘Often/Always’) and ‘Refused/Don’t know. ‘Refused/don’t know’ category is not included in modelling. |
| **Interview reported after Omicron variants became dominant** | Categorization of follow-up interview date as being before December 31 2021 or on or after December 31 2021. The Omicron variant became dominant (>99%) by the end of December, so the end of December was selected to ensure individuals identified as reporting ‘during Omicron’ were, if infected, almost certainly infected with this variant |
| **Proportion of interval spent in congregate (shared) homeless shelter** | Proportion of the interval spent by the participant in congregate (shared sleeping space) homeless shelter(s). Minimum value = 0%; Maximum value = 100% |
| **Proportion of interval spent in non-congregate (private) homeless shelter** | Proportion of the interval spent by the participant in non-congregate (private sleeping space) homeless shelter(s). Minimum value = 0%; Maximum value = 100% |
| **Proportion of interval spent in physical distancing hotel** | Proportion of the interval spent by the participant in physical distancing hotel(s). Minimum value = 0%; Maximum value = 100% |
| **Proportion of interval spent in own place** | Proportion of the interval spent by the participant in their own home/accommodation (ie. The participant is ‘housed’). Minimum value = 0%; Maximum value = 100% |
| **Proportion of interval spent staying with friends or family** | Proportion of the interval spent by the participant staying with friends or family. Minimum value = 0%; Maximum value = 100% |
| **Proportion of interval spent in High Exposure setting** | Proportion of the interval spent by the participant in housing settings considered ‘high exposure’, ie. At highest risk of contracting SARS-CoV-2 due to crowding, turnover and/or congregate nature of sleeping/living setting(s). Includes the following housing types: congregate homeless shelter, recovery centre, nursing home, jail or immigration detention centre. Minimum value = 0%; Maximum value = 100% |
| **Proportion of interval spent in Moderate Exposure setting** | Proportion of the interval spent by the participant in housing settings considered ‘moderate exposure’, ie. At less risk than ‘High exposure’ and greater risk than ‘Low exposure’ of contracting SARS-CoV-2 due to crowding, turnover and/or congregate nature of sleeping/living setting(s). Includes the following housing types: physical distancing hotel, non-congregate shelter, transitional housing, rooming house, encampment, on the street, rehab, hospital or 'other' settings. Minimum value = 0%; Maximum value = 100% |
| **Proportion of interval spent in Low Exposure setting** | Proportion of the interval spent by the participant in housing settings considered ‘low exposure’, ie. At least risk of contracting SARS-CoV-2 due to lower crowding, turnover and/or less congregate nature of sleeping/living setting(s). Includes the following housing types: own home, supportive housing, private hotel/motel, or staying with friends and family. Minimum value = 0%; Maximum value = 100% |
| **Number of moves during the interval** | Number of housing moves by the participant during the interval. Includes changes in residence (new housing episodes) as well as moves indicated by the participant during periods where housing was shared between two or more locations. |
| **Average number of people who shared living space** | Number of people sharing living space with the participants during the interval, on average. Reported in the housing history for each housing episode, this may thus represent the ‘average of an average’ where number with whom living space is shared fluctuated within a housing episode (for example, in a congregate homeless shelter) as well as between episodes. |

**Supplement C - Supplement tables**

**Supplement C, table 1: *Ku-gaa-gii pimitizi-win* self-reported characteristics, overall vs successfully linked to ICES**

|  | **Total (N=736)** | **Linked at ICES (N=640)** |
| --- | --- | --- |
| Age category, N (%) |  |  |
| 16 to 29 years | 100 (13.59%) | 74 (11.56%) |
| 30 to 49 years | 326 (44.29%) | 280 (43.75%) |
| 50 to 69 years | 263 (35.73%) | 242 (37.81%) |
| 70+ years | 47 (6.39%) | 44 (6.88%) |
| Self-reported Gender, N (%) |  |  |
| Male | 486 (66.03%) | 438 (68.44%) |
| Female | 231 (31.39%) | 185 (28.91%) |
| LGBTQ2+/Non-binary/Other | 17 (2.31%) | NR |
| Missing | 2 (0.27%) | <=5 |
| Self-identify as Indigenous, N (%) | 76 (10.23%) | 72 (11.25%) |
| Self-reported race category, N (%) |  |  |
| White | 359 (48.78%) | 332 (51.88%) |
| Black | 159 (21.60%) | 122 (19.06%) |
| Indigenous | 28 (3.80%) | 28 (4.38%) |
| Other/multiracial | 157 (21.33%) | 134 (20.94%) |
| Refused/Don't know | 33 (4.48%) | 24 (3.75%) |
| Citizenship status, N (%) |  |  |
| Citizen | 564 (76.63%) | 520 (81.25%) |
| Landed immigrant/Permanent Resident | 90 (12.23%) | 84 (13.13%) |
| Refugee claimant | 55 (7.47%) | 24 (3.75%) |
| Temporary/Other | 20 (2.72%) | NR |
| Refused/Don't know | 7 (0.95%) | <=5 |
| Immigrated to Canada within the past 10 years, N (%) |  |  |
| N/A (born in Canada) | 441 (59.92%) | 404 (63.13%) |
| No, immigrated > 10 years ago | 187 (25.41%) | 172 (26.88%) |
| Yes, immigrated <=10 years ago | 108 (14.67%) | 64 (10.0%) |
| Highest level of education completed, N (%) |  |  |
| Have not completed High School | 208 (28.26%) | 188 (29.38%) |
| High School or Secondary School | 255 (34.65%) | 223 (34.84%) |
| Any post-secondary | 264 (35.87%) | 223 (34.84%) |
| Refused/Don't know | 9 (1.22%) | 6 (0.94%) |
| BMI category, N (%) |  |  |
| Underweight/Normal | 339 (46.06%) | 301 (47.03%) |
| Overweight | 227 (30.84%) | 199 (31.09%) |
| Obese | 141 (19.16%) | 119 (18.59%) |
| Missing | 29 (3.94%) | 21 (3.28%) |
| Confidence of interviewer in responses received during interview, N (%) |  |  |
| Confident | 715 (97.15%) | 623 (97.34%) |
| Unconfident | 21 (2.85%) | 17 (2.66%) |

**Supplement table 2: Participant and interval characteristics by outcome status (2,401 intervals overall)**

|  | **Intervals resulting in no SARS-CoV-2 infection (n=1,840)** | **Intervals resulting in SARS-CoV-2 infection (n=561)** |  |
| --- | --- | --- | --- |
| **Socio-demographic characteristics** | | |  |
| Age category at baseline, N (%) |  |  |  |
| 16-29 years old | | 190 (10.3%) | 60 (10.7%) |
| 30-49 years old | | 748 (40.7%) | 249 (44.4%) |
| 50-69 years old | | 760 (41.3%) | 210 (37.4%) |
| 70+ years old | | 142 (7.7%) | 42 (7.5%) |
| Self-reported gender, N (%) |  |  |  |
| Male | 1,223 (66.5%) | 403 (71.8%) |  |
| Female | 575 (31.3%) | 148 (26.4%) |  |
| LGBTQS2+/Non-binary/Other | <=50 | <=10 |  |
| Refused/Don’t know | <=5 | <=5 |  |
| Self-reported race category, N (%) |  |  |  |
| White | 996 (54.1%) | 270 (48.1%) |  |
| Black | 327 (17.8%) | 120 (21.4%) |  |
| Indigenous | 77 (4.2%) | 25 (4.5%) |  |
| Other/Multiracial | 369 (20.1%) | 127 (22.6%) |  |
| Refused/Don’t know | 71 (3.9%) | 19 (3.4%) |  |
| Citizenship status, N (%) |  |  |  |
| Citizen | 1,508 (82.0%) | 445 (79.3%) |  |
| Landed immigrant/permanent resident | 237 (12.9%) | 83 (14.8%) |  |
| Refugee claimant | 71 (3.9%) | 22 (3.9%) |  |
| Temporary status/Other | <=30 | <=10 |  |
| Refused/Don’t know | <=5 | <=5 |  |
| Immigration history, N (%) |  |  |  |
| Born in Canada | 513 (27.9%) | 168 (29.9%) |  |
| Immigrated to Canada > 10 years ago | 166 (9.0%) | 61 (10.9%) |  |
| Immigrated to Canada <= 10 years ago | 1,161 (63.1%) | 332 (59.2%) |  |
| Education completed, N (%) |  |  |  |
| Less than high school | 534 (29.0%) | 175 (31.2%) |  |
| High school or equivalent | 650 (35.3%) | 197 (35.1%) |  |
| Any post-secondary | 656 (35.7%) | 189 (33.7%) |  |
| **Health status at baseline** | | |  |
| Hypertension, N (%) | 330 (17.9%) | 105 (18.7%) |  |
| Diabetes, N (%) | 272 (14.8%) | 77 (13.7%) |  |
| Asthma, N (%) | 363 (19.7%) | 111 (19.8%) |  |
| Chronic Lung Disease , N (%) | 301 (16.4%) | 80 (14.3%) |  |
| Chronic Heart Disease, N (%) | 81 (4.4%) | 22 (3.9%) |  |
| History of Stroke, N (%) | 42 (2.3%) | 15 (2.7%) |  |
| Chronic Kidney Disease, N (%) | 35 (1.9%) | 10 (1.8%) |  |
| Chronic Neurological Disorder, N (%) | 84 (4.6%) | 26 (4.6%) |  |
| Liver Disease, N (%) | 26 (1.4%) | 15 (2.7%) |  |
| Cancer diagnosis within the past ten years N (%) | 39 (2.1%) | 16 (2.9%) |  |
| HIV/AIDS, N (%) | 33 (1.8%) | 11 (2.0%) |  |
| Any mental health or substance use related disorder, N (%) |  |  |  |
| Any | 764 (41.5%) | 240 (42.8%) |  |
| Substance use disorders | 244 (13.3%) | 84 (15.0%) |  |
| Psychotic disorders including schizophrenia | 144 (7.8%) | 50 (8.9%) |  |
| Mood and anxiety disorders | 278 (15.1%) | 86 (15.3%) |  |
| OCD/Personality disorders | 25 (1.4%) | 17 (3.0%) |  |
| Intentional self-injury | 73 (4.0%) | 25 (4.5%) |  |
| COVID-19 infections, N (%) |  |  |  |
| None | 1,436 (78.0%) | 260 (46.3%) |  |
| 1 infection | 373 (20.3%) | 282 (50.3%) |  |
| 2+ infections | 31 (1.7%) | 19 (3.4%) |  |
| COVID-19 vaccines, N (%) |  |  |  |
| None | 399 (21.7%) | 139 (24.8%) |  |
| Incomplete primary series | 295 (16.0%) | 98 (17.5%) |  |
| Full primary series | 1,146 (62.3%) | 324 (57.8%) |  |
| **Health-related behaviours reported at interview** | | |  |
| Paid or volunteer work, N (%) | 501 (27.2%) | 158 (28.2%) |  |
| Alcohol consumption, N (%) | 1,066 (57.9%) | 357 (63.6%) |  |
| Alcohol consumption frequency, N (%) |  |  |  |
| Never | 767 (41.7%) | 200 (35.7%) |  |
| Monthly or less | 416 (22.6%) | 143 (25.5%) |  |
| 2-4 times a month | 256 (13.9%) | 77 (13.7%) |  |
| 2-3 times a week | 188 (10.2%) | 69 (12.3%) |  |
| 4+ times a week | 213 (11.6%) | 72 (12.8%) |  |
| Tobacco consumption, N (%) | 1,268 (68.9%) | 369 (65.8%) |  |
| Tobacco consumption frequency, N (%) |  |  |  |
| Never | 569 (30.9%) | 190 (33.9%) |  |
| Less than daily | 1,078 (58.6%) | 320 (57.0%) |  |
| Daily | 193 (10.5%) | 51 (9.1%) |  |
| Consumption of illegal/prescription medication for non-medical reasons, N (%) | 514 (27.9%) | 174 (31.0%) |  |
| PHG #1: wears face mask in public, N (%) |  |  |  |
| Good (Often or always) | 1,488 (80.9%) | 460 (82.0%) |  |
| Poor (Never, Rarely or Occasionally) | 352 (19.1%) | 101 (18.0%) |  |
| PHG #2: distances in public places, N (%) |  |  |  |
| Good (Often or always) | 1,526 (82.9%) | 462 (82.4%) |  |
| Poor (Never, Rarely or Occasionally) | 314 (17.1%) | 99 (17.6%) |  |
| PHG #3: avoids crowded places or gatherings, N (%) |  |  |  |
| Good (Often or always) | 1,417 (77.0%) | 428 (76.3%) |  |
| Poor (Never, Rarely or Occasionally) | 423 (23.0%) | 133 (23.7%) |  |
| Interview timing, N (%) |  |  |  |
| Pre-Omicron (<=31 Dec 2021) | 912 (49.6%) | 260 (46.3%) |  |
| Post-Omicron (>31 Dec 2021) | 928 (50.4%) | 301 (53.7%) |  |
| Proportion of interval spent in : |  |  |  |
| Congregate shelter (mean, SD) | 23.67 ± 39.80 | 29.93 ± 42.70 |  |
| Non-congregate shelter (mean, SD) | 9.86 ± 28.29 | 9.13 ± 26.82 |  |
| Physical distancing hotel (mean, SD) | 43.20 ± 46.98 | 38.29 ± 45.37 |  |
| Own place (mean, SD) | 15.05 ± 33.57 | 11.25 ± 28.82 |  |
| Staying with friends or family (mean, SD) | 4.08 ± 16.18 | 3.84 ± 15.34 |  |
|  |  |  |  |
| High Exposure^a^ setting (mean, SD) | 24.00 ± 39.97 | 31.57 ± 43.27 |  |
| Moderate Exposure^b^ setting (mean, SD) | 56.72 ± 46.37 | 52.50 ± 46.29 |  |
| Low Exposure^c^ setting (mean, SD) | 17.39 ± 34.52 | 14.35 ± 31.18 |  |
| Majority of interval spent in…, N (%) |  |  |  |
| High Exposure^a^ setting | 448 (24.3%) | 180 (32.1%) |  |
| Moderate Exposure^b^ setting | 1,041 (56.6%) | 300 (53.5%) |  |
| Low Exposure^c^ setting | 351 (19.1%) | 81 (14.4%) |  |
| Number of moves during period, mean (SD) | 0.68 ± 1.12 | 0.90 ± 1.34 |  |
| Average number of people who shared living space, mean (SD) | 4.68 ± 11.88 | 7.19 ± 17.70 |  |

SD=Standard Deviation; PHG=Public Health Guideline

^a^ ’High exposure’ includes time residing in a congregate homeless shelter, recovery centre, nursing home, jail or immigration detention center

^b^ ’Moderate exposure’ includes time residing in a physical distancing hotel, non-congregate shelter, transitional housing, rooming house, encampment, on the street, rehab, hospital or 'other' settings.

^c^ ’Low exposure’ includes time residing in own home, supportive housing, private hotel/motel, or staying with friends and family

**Supplement D: The RECORD statement^1^ – checklist of items, extended from the STROBE statement, that should be reported in observational studies using routinely collected health data.**

|  | **Item No.** | **STROBE items** | **Location in manuscript where items are reported** | **RECORD items** | **Location in manuscript where items are reported** |
| --- | --- | --- | --- | --- | --- |
| **Title and abstract** | | | | | |
|  | 1 | (a) Indicate the study’s design with a commonly used term in the title or the abstract (b) Provide in the abstract an informative and balanced summary of what was done and what was found |  | RECORD 1.1: The type of data used should be specified in the title or abstract. When possible, the name of the databases used should be included.  RECORD 1.2: If applicable, the geographic region and timeframe within which the study took place should be reported in the title or abstract.  RECORD 1.3: If linkage between databases was conducted for the study, this should be clearly stated in the title or abstract. | Title; Abstract |
| **Introduction** | | | | | |
| Background rationale | 2 | Explain the scientific background and rationale for the investigation being reported |  |  | Introduction |
| Objectives | 3 | State specific objectives, including any prespecified hypotheses |  |  | Introduction, final paragraph |
| **Methods** | | | | | |
| Study Design | 4 | Present key elements of study design early in the paper |  |  | Methods; Study design and setting |
| Setting | 5 | Describe the setting, locations, and relevant dates, including periods of recruitment, exposure, follow-up, and data collection |  |  | Methods; Study design and setting |
| Participants | 6 | *(a) Cohort study* - Give the eligibility criteria, and the sources and methods of selection of participants. Describe methods of follow-up  *Case-control study* - Give the eligibility criteria, and the sources and methods of case ascertainment and control selection. Give the rationale for the choice of cases and controls  *Cross-sectional study* - Give the eligibility criteria, and the sources and methods of selection of participants  *(b) Cohort study* - For matched studies, give matching criteria and number of exposed and unexposed  *Case-control study* - For matched studies, give matching criteria and the number of controls per case |  | RECORD 6.1: The methods of study population selection (such as codes or algorithms used to identify subjects) should be listed in detail. If this is not possible, an explanation should be provided.  RECORD 6.2: Any validation studies of the codes or algorithms used to select the population should be referenced. If validation was conducted for this study and not published elsewhere, detailed methods and results should be provided.  RECORD 6.3: If the study involved linkage of databases, consider use of a flow diagram or other graphical display to demonstrate the data linkage process, including the number of individuals with linked data at each stage. | 6.1 Methods; Data Sources; Recruitment and follow-up  6.2 Methods; Covariates; Supplement 2  6.3 Figure 1 |
| Variables | 7 | Clearly define all outcomes, exposures, predictors, potential confounders, and effect modifiers. Give diagnostic criteria, if applicable. |  | RECORD 7.1: A complete list of codes and algorithms used to classify exposures, outcomes, confounders, and effect modifiers should be provided. If these cannot be reported, an explanation should be provided. | Supplement 1 and 2 |
| Data sources/ measurement | 8 | For each variable of interest, give sources of data and details of methods of assessment (measurement).  Describe comparability of assessment methods if there is more than one group |  |  | Methods: Data sources, Covariates, Outcomes, Statistical Analysis  Supplement 2 |
| Bias | 9 | Describe any efforts to address potential sources of bias |  |  | N/A |
| Study size | 10 | Explain how the study size was arrived at |  |  | N/A (Referred reader to protocol which features sample size calculation) |
| Quantitative variables | 11 | Explain how quantitative variables were handled in the analyses. If applicable, describe which groupings were chosen, and why |  |  | Methods: Covariates and Supplement 2 |
| Statistical methods | 12 | (a) Describe all statistical methods, including those used to control for confounding  (b) Describe any methods used to examine subgroups and interactions  (c) Explain how missing data were addressed  (d) *Cohort study* - If applicable, explain how loss to follow-up was addressed  *Case-control study* - If applicable, explain how matching of cases and controls was addressed  *Cross-sectional study* - If applicable, describe analytical methods taking account of sampling strategy  (e) Describe any sensitivity analyses |  |  | Methods, Statistical Analysis |
| Data access and cleaning methods |  | .. |  | RECORD 12.1: Authors should describe the extent to which the investigators had access to the database population used to create the study population.  RECORD 12.2: Authors should provide information on the data cleaning methods used in the study. | 12.1 Noted work at ICES in Methods: Study Design & Setting  12.2 N/A |
| Linkage |  | .. |  | RECORD 12.3: State whether the study included person-level, institutional-level, or other data linkage across two or more databases. The methods of linkage and methods of linkage quality evaluation should be provided. | Methods: Study Design and Setting |
| **Results** | | | | | |
| Participants | 13 | (a) Report the numbers of individuals at each stage of the study (*e.g.*, numbers potentially eligible, examined for eligibility, confirmed eligible, included in the study, completing follow-up, and analysed)  (b) Give reasons for non-participation at each stage.  (c) Consider use of a flow diagram |  | RECORD 13.1: Describe in detail the selection of the persons included in the study (*i.e.,* study population selection) including filtering based on data quality, data availability and linkage. The selection of included persons can be described in the text and/or by means of the study flow diagram. | 13.1 Figure 1; Results paragraph 1 |
| Descriptive data | 14 | (a) Give characteristics of study participants (*e.g.*, demographic, clinical, social) and information on exposures and potential confounders  (b) Indicate the number of participants with missing data for each variable of interest  (c) *Cohort study* - summarise follow-up time (*e.g.*, average and total amount) |  |  | Results, Paragraph 1 |
| Outcome data | 15 | *Cohort study* - Report numbers of outcome events or summary measures over time  *Case-control study* - Report numbers in each exposure category, or summary measures of exposure  *Cross-sectional study* - Report numbers of outcome events or summary measures |  |  | Results, Paragraph 2 |
| Main results | 16 | (a) Give unadjusted estimates and, if applicable, confounder-adjusted estimates and their precision (e.g., 95% confidence interval). Make clear which confounders were adjusted for and why they were included  (b) Report category boundaries when continuous variables were categorized  (c) If relevant, consider translating estimates of relative risk into absolute risk for a meaningful time period |  |  | Results, Paragraph 2, 3, 4 |
| Other analyses | 17 | Report other analyses done—e.g., analyses of subgroups and interactions, and sensitivity analyses |  |  | N/A |
| **Discussion** | | | | | |
| Key results | 18 | Summarise key results with reference to study objectives |  |  | Discussion paragraph 1,2 |
| Limitations | 19 | Discuss limitations of the study, taking into account sources of potential bias or imprecision. Discuss both direction and magnitude of any potential bias |  | RECORD 19.1: Discuss the implications of using data that were not created or collected to answer the specific research question(s). Include discussion of misclassification bias, unmeasured confounding, missing data, and changing eligibility over time, as they pertain to the study being reported. | Discussion, Limitations section |
| Interpretation | 20 | Give a cautious overall interpretation of results considering objectives, limitations, multiplicity of analyses, results from similar studies, and other relevant evidence |  |  | Discussion, paragraph 1 and 2 |
| Generalisability | 21 | Discuss the generalisability (external validity) of the study results |  |  | Discussion, paragraph 2 and Limitations section |
| **Other Information** | | | | | |
| Funding | 22 | Give the source of funding and the role of the funders for the present study and, if applicable, for the original study on which the present article is based |  |  | Acknowledgements, funding statement |
| Accessibility of protocol, raw data, and programming code |  | .. |  | RECORD 22.1: Authors should provide information on how to access any supplemental information such as the study protocol, raw data, or programming code. |  |

*Checklist is protected under Creative Commons Attribution ([CC BY](http://creativecommons.org/licenses/by/4.0/)) license.

**References**

1. Gershon AS, Wang C, Guan J, Vasilevska-Ristovska J, Cicutto L, To T. Identifying patients with physician-diagnosed asthma in health administrative databases. Canadian Respiratory Journal. 2009 Nov 1;16:183-8.
2. Gershon AS, Wang C, Guan J, Vasilevska-Ristovska J, Cicutto L, To T. Identifying individuals with physcian diagnosed COPD in health administrative databases. COPD: Journal of Chronic Obstructive Pulmonary Disease. 2009 Jan 1;6(5):388-94.
3. Guttmann A, Nakhla M, Henderson M, To T, Daneman D, Cauch‐Dudek K, Wang X, Lam K, Hux J. Validation of a health administrative data algorithm for assessing the epidemiology of diabetes in Canadian children. Pediatric diabetes. 2010 Mar;11(2):122-8.
4. Schultz SE, Rothwell DM, Chen Z, Tu K. Identifying cases of congestive heart failure from administrative data: a validation study using primary care patient records. Chronic diseases and injuries in Canada. 2013 Jun 1;33(3).
5. Tu K, Campbell NR, Chen ZL, Cauch-Dudek KJ, McAlister FA. Accuracy of administrative databases in identifying patients with hypertension. Open medicine. 2007;1(1):e18.
6. Antoniou T, Zagorski B, Loutfy MR, Strike C, Glazier RH. Validation of case-finding algorithms derived from administrative data for identifying adults living with human immunodeficiency virus infection. PloS one. 2011 Jun 30;6(6):e21748.
7. Richard L, Nisenbaum R, Colwill K, Mishra S, Dayam RM, Liu M, Pedersen C, Gingras AC, Hwang SW. Enhancing detection of SARS-CoV-2 re-infections using longitudinal sero-monitoring: demonstration of a methodology in a cohort of people experiencing homelessness in Toronto, Canada. *BMC Infectious Diseases*. 2024 Feb 2; 24(1): 125.
8. Benchimol EI, Smeeth L, Guttmann A, Harron K, Moher D, Petersen I, Sørensen HT, von Elm E, Langan SM, RECORD Working Committee. The REporting of studies Conducted using Observational Routinely-collected health Data (RECORD) statement. PLoS medicine. 2015 Oct 6;12(10):e1001885.
